# Transcranial Doppler Can Predict Development and Outcome of Sepsis-Associated Encephalopathy in Pediatrics With Severe Sepsis or Septic Shock

**DOI:** 10.3389/fped.2020.00450

**Published:** 2020-08-20

**Authors:** HebatAllah Algebaly, Seham ElSherbini, Ahmed Galal, Rania Hamdi, Ahmed Baz, Ahmed Elbeleidy

**Affiliations:** ^1^Pediatric Critical Care Unit, Department of Pediatrics, Cairo University, Cairo, Egypt; ^2^Pediatric Critical Care Unit, Children's Cancer Hospital, Cairo University, Cairo, Egypt; ^3^Pediatric Imaging Unit, Department of Radiology, Cairo University, Cairo, Egypt

**Keywords:** septic shock, cerebrovascular resistance, pulsatility index, septic encephalopathy, transcranial Doppler

## Abstract

**Background and Aim:** Sepsis is a common cause of pediatric intensive care unit (ICU) admission. Sepsis-associated encephalopathy (SAE) may occur owing to brain dysfunction in those patients and may be related to impaired cerebral microcirculation. Transcranial Doppler (TCD) can be used to detect this impairment. In this study, we aimed to assess the role of TCD in prediction of SAE and mortality in patients with severe sepsis or septic shock admitted to PICU.

**Patients and Methods:** This prospective study included 75 children admitted to PICU owing to severe sepsis or septic shock. Upon admission, all patients were subjected to careful history taking, thorough clinical examination, and standard laboratory workup. Severity of clinical illness was assessed using the Pediatric Risk of Mortality (PRISM) III score. TCD was performed on the first day of admission after the normalization of systolic blood pressure with or without vasopressors. The primary study outcome was differences in the measurement of TCD in SAE, and the secondary outcome was discharge from ICU or mortality.

**Results:** The study comprised 45 children with SAE and 30 age- and sex-matched children without SAE. In this study, SAE patients had significantly higher pulsatility index [PI; median interquartile range (IQR): 1.15 (0.98–1.48) vs. 1.0 (0.95–1.06), *p* = 0.002] and resistive index [RI; median (IQR): 0.68 (0.61–0.77) vs. 0.62 (0.59–0.64), *p* = 0.001] than had non-SAE patients. PI and RI showed good performance as predictors of subsequent SAE development [area under the curve (AUC): 0.72 and 0.73, respectively]. Non-survivors in SAE patients had significantly higher PRISM III. Receiver operating characteristic (ROC) curve analysis showed good performance of PI and RI as predictors of mortality at the end of follow-up.

**Conclusions:** In children with SAE, cerebrovascular resistance is high and is associated with increased mortality.

## Introduction

Sepsis contributes to 70% of pediatric intensive care unit (PICU) admission in developing countries (1). Brain dysfunction is one of the most critical sequelae of sepsis and may manifest as sepsis-associated encephalopathy (SAE). The incidence of septic brain disease was 8–70% (2). The clinical spectrum of SAE ranges from mild disorientation or agitation to severe disturbance of consciousness with no direct infection or invasion of the central nervous system (3). Patients with SAE show high risk of mortality (4). Wilson and Young (5) redefined the brain dysfunction caused by sepsis as septic-related encephalopathy, which was a very common disease in PICU with a high mortality rate and high hospitalization cost and was often seen in children. Pathogenesis of septic encephalopathy in children remains unclear (6). Vasomotor dysfunction, which was regarded as the main cause of SAE, often leads to dysregulation of cerebral blood flow and can directly affect brain function and cerebral blood circulation (7).

Impaired cerebral microcirculation and disturbed cerebral blood flow were suggested as possible mechanisms of SAE (8, 9). This may be related to the increased local production of endothelin-1 among other mediators within the cerebral vasculature in the context of the exaggerated systemic inflammatory response associated with sepsis (10).

Bedside transcranial Doppler (TCD) may provide important diagnoses and prognoses about cerebrovascular hemodynamics in children with various disorders, for example, traumatic brain injury, intracranial hypertension, vasospasm, stroke, cerebrovascular disorders, central nervous system infections, and brain death (11). Three main parameters can be obtained from the Doppler spectrum: flow direction, velocities, and indices for arterial resistance. The latter includes the pulsatility index (PI) and the resistive index (RI) (12). PI was reported to increase during adult sepsis (7, 13–18).

In this study, we aimed to assess the role of TCD in prediction of SEA and mortality in patients with severe sepsis or septic shock admitted to PICU.

## Methodology

This prospective study was conducted at Cairo University Hospital, Cairo, Egypt. The study protocol was approved by the local ethical committee, and the legal guardians of the included children gave informed consent on admission.

We included two groups: the first group comprised children in severe sepsis or septic shock with encephalopathy after confirmation of normal cerebrospinal fluid analysis, absence of primary brain pathology in head computerized tomography, or any metabolic disturbance such as electrolyte disorders, uremia, or hyperammonemia in the emergency department and prior to PICU admission. This is to eliminate the possibility of infectious meningitis, encephalitis, or encephalopathy secondary to causes other than sepsis.

The other group comprised critically sick children with no encephalopathy. Sepsis was defined and classified according to the recommendations of the International Consensus Conference 2005 (19). SAE was suspected in patients presenting with acutely altered mental status accompanied by sepsis or septic shock (20). In the encephalopathic group, we included severe sepsis or septic shock children with any degree of disturbed conscious level from drowsiness to coma. Full Outline of Unresponsiveness (FOUR) score was <16. FOUR score is a coma scale that consists of four components (eye response, motor response, brain stem reflexes, and respiration) (21).

Upon admission, all patients were subjected to careful history taking, thorough clinical examination, and standard laboratory workup. Severity of clinical illness was assessed using the Pediatric Risk of Mortality (PRISM) III score (22).

Neuroimaging studies were done to exclude cerebrovascular events. TCD was performed on the first day of admission after the normalization of systolic arterial blood pressure with or without vasopressors. We used the percentiles of systolic blood pressure to achieve a minimum of the fifth centile for age (23).

An ultrasound machine (GE Logiq® P3) manufactured in USA with a 3-MHz TCD probe was used. The probe was directed through the temporal bone window on both sides of the skull. The temporal window was used to examine the middle cerebral artery. PI and RI were calculated as follows: PI = systolic velocity – (diastolic velocity/mean velocity); RI = systolic velocity – (diastolic velocity/systolic velocity). Every measurement on each side of the brain was repeated three times, and the highest value was considered for the analysis. The average of the two values for the two brain sides was registered. The primary study outcome was the difference in the values of PI and RI between the septic encephalopathy children and fully conscious non-septic critical children, and the secondary outcome was discharge from ICU or mortality. The images were recorded by the intensivist and reviewed by the radiologist for interpretation. The intensivist received extensive training on proper image acquisition by the pediatric radiologist. Sedation with midazolam was part of the management of ventilated patients to help reduce the intracranial pressure, and the FOUR score (21) was calculated just before starting midazolam infusion.

Data were analyzed using the Statistical Package for Social Sciences (SPSS) software, version 21 (SPSS; IBM, Chicago, IL 60606, USA). Data were expressed as mean and standard deviations or median and interquartile range (IQR) for quantitative variables, and frequencies and percentages for qualitative variables. Comparisons between groups were performed using an independent samples *t*-test or Mann–Whitney *U*-test for quantitative variables and the chi-square or Fisher exact test for qualitative variables. Receiver operating characteristic (ROC) curve analysis was used to identify predictive performance of PI and RI. *p* < 0.05 were considered statistically significant.

## Result

**Figure 1:**
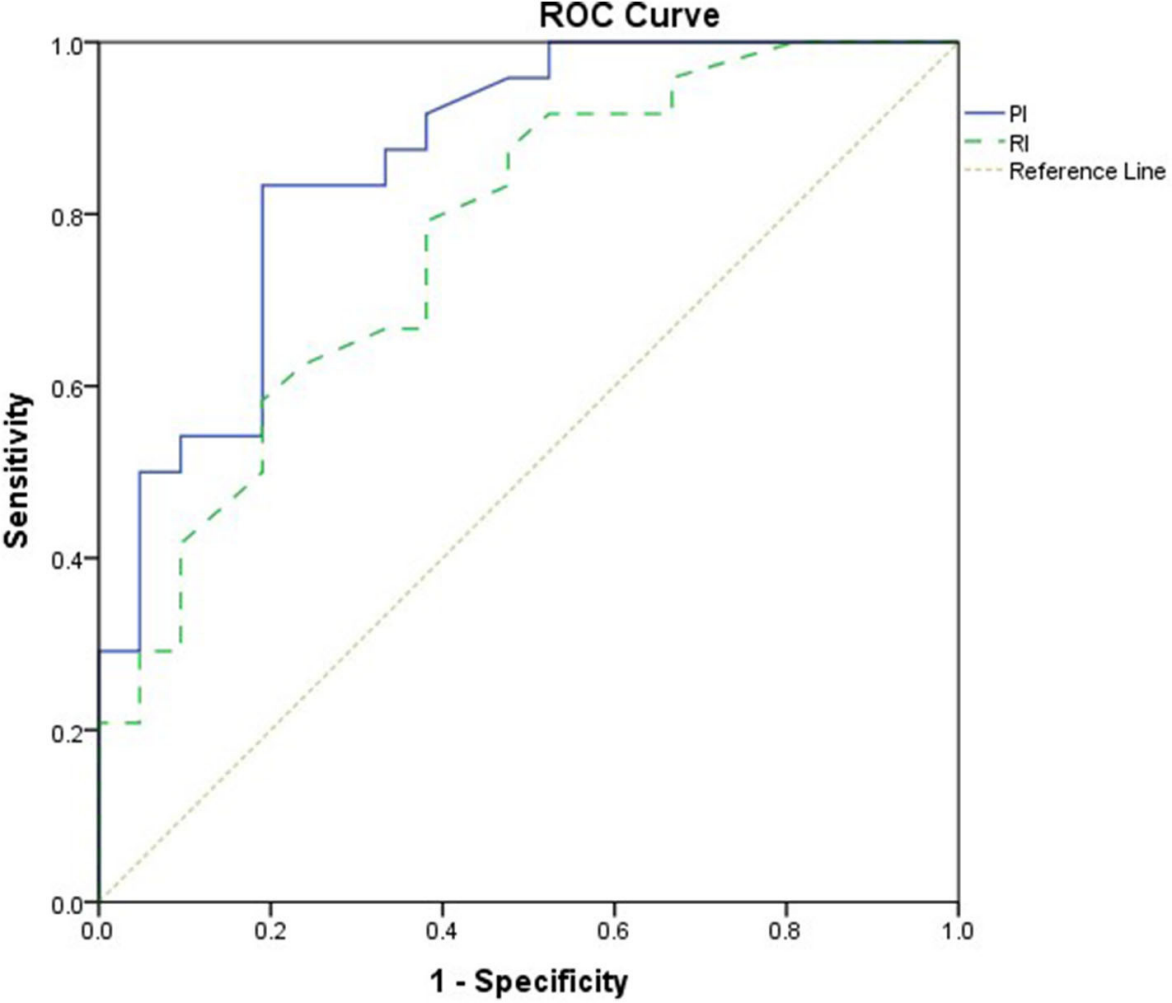
Receiver operating characteristic (ROC) curve analysis showed good performance of pulsatility index (PI) and resistive index (RI) as predictors of subsequent sepsis-associated encephalopathy (SAE).

**Figure 2:**
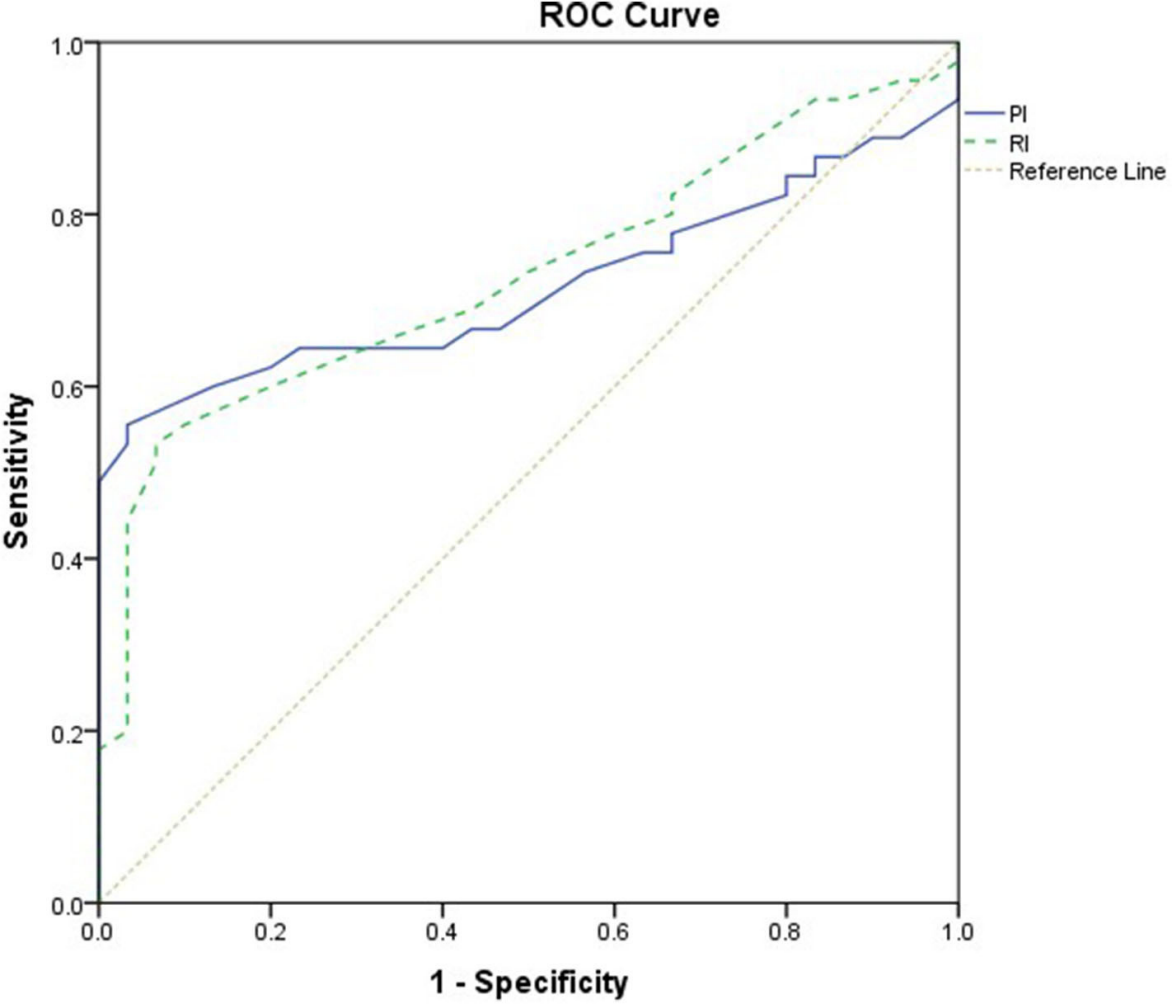
Receiver operating characteristic (ROC) curve analysis showed good performance of pulsatility index (PI) and resistive index (RI) as predictors of mortality.

This study included 45 children with SAE and 30 age- and sex-matched critically sick children without encephalopathy from the PICU of a tertiary hospital. All of them are previously healthy children. The diagnoses are in Table 1. The control group included Guillain–Barre syndrome, bronchiolitis, bronchial asthma, postoperative abdominal surgery, congenital heart disease, and acute hemolytic anemia. Comparison between the studied groups regarding the clinical data and outcome parameters revealed significantly higher PRISM III score in encephalopathic patients (20.0 ± 6.0 vs. 6.8 ± 1.0, *p* < 0.001). They also had significantly higher frequency of vasopressor use, mechanical ventilation, and deceased patients at the end of follow-up. Moreover, it was found that encephalopathic patients had significantly higher PI [median (IQR): 1.15 (0.98–1.48) vs. 1.0 (0.95–1.06), *p* = 0.002] and RI [median (IQR): 0.68 (0.61–0.77) vs. 0.62 (0.59–0.64), *p* = 0.001] than had non-encephalopathic patients. PI and RI showed good performance as predictors of subsequent SAE development [area under the curve (AUC): 0.72 and 0.73, respectively] (Tables 1, 2, Figure 1). The correlation analysis revealed significant direct correlation between PI and PRISM III score and significant inverse correlation between PI and FOUR score in encephalopathic patients (Table 3). The comparison between survivors and non-survivors in the encephalopathic group showed that non-survivors had significantly higher PRISM III and FOUR scores. They also had significantly higher frequency of cases with mechanical ventilation and shorter duration of ICU stay. In addition, they had significantly higher PI and RI at baseline than had survivors (Table 4). ROC curve analysis showed good performance of PI and RI as predictors of mortality at the end of follow-up (Table 5, Figure 2). PRISM score, use of vasopressors, PI, and RI were significant predictors of SAE in the univariate analysis. However, in the multivariate analysis only PRISM score, PI and RI remained significant (Table 6). PRISM score, FOUR score, PI, and RI were significant predictors of mortality in the univariate analysis. However, in the multivariate analysis, only PRISM score and RI remained significant Table 7).

**Table 1:** Clinical and outcome parameters in the studied patients.

	**SAE patients** ***n* = 45**	**Non-SAE patients** ***n* = 30**	***p* value**
Age (months) median (IQR)	10.0 (6.0–30.0)	12.5 (6.0–22.5)	0.89
Male/female, *n*	26/19	20/10	0.439
**Sepsis or septic shock focus**, ***n*** **(%)**			
Intestinal perforation	4 (8.9)	0	0.96
Pneumonia	17 (37.8)		
Blood borne	65 (11%)		
Endocarditis	5 (11.1)		
Diarrhea	14 (31.1)		
**Etiology of critical disease in non-encephalopathic children**			
Guillain–Barre syndrome		7 (10%)	
Bronchiolitis		14 (50%)	
Acute hemolytic anemia		3 (4%)	
Congenital heart disease		3 (4%)	
PRISM score, mean ± SD	20.0 ± 6.0	6.8 ± 1.0	<0.001
PI median (IQR)	1.15 (0.98–1.48)	1.0 (0.95–1.06)	0.002
RI median (IQR)	0.68 (0.61–0.77)	0.62 (0.59–0.64)	0.001
Use of vasopressors, *n* (%)	33 (73.3)	–	<0.001
Mechanical ventilation, *n* (%)	36 (80.0)	17 (56.7)	0.03
ICU stay (days), mean ± SD	14.0 ± 1.9	5.0 ± 1.0	<0.001
Mortality, *n* (%)	24 (53.3)	–	<0.001

*PI, pulsatility index; RI, resistive index; ICU, intensive care unit; SAE, sepsis-associated encephalopathy; IQR, interquartile range; PRISM, Pediatric Risk of Mortality*.

**Table 2:** Value of PI and RI in predicting SAE.

	**PI**	**RI**
Cutoff	1.0	0.62
AUC	0.72 (95.0% CI: 0.6–0.83)	0.73 (95.0% CI: 0.62–0.84)
*p*	0.002	0.001
Sensitivity	73.3%	73.3%
Specificity	43.3%	50.0

*PI, pulsatility index; RI, resistive index; AUC, area under the curve; SAE, sepsis-associated encephalopathy*.

**Table 3:** Correlation between PI and RI and other clinical data in encephalopathic patients.

	**PI**		**RI**	
	***r***	***p***	***R***	***p***
Age	−0.112	0.46	−0.23	0.12
PRISM III score	0.340	0.02	0.282	0.06
FOUR score	−0.357	<0.01	−0.275	0.06
ICU stay	−0.03	0.85	−0.01	0.93
Mechanical ventilation	Mean ± SD	*p*	Mean ± SD	*p*
Yes	1.3 ± 0.07	0.001	0.7 ± 0.018	0.34
No	1.05 ± 0.019		0.64 ± 0.2	

*PI, pulsatility index; RI, resistive index; ICU, intensive care unit; PRISM III, pediatric risk of mortality assessment; FOUR score, Full Outline of Unresponsiveness score*.

**Table 4:** Comparison between survivors and non-survivors at ICU discharge.

	**Non-survivors *n* = 24**	**Survivors *n* = 21**	***p* value**
Age (months) median (IQR)	12.0 (6.0–36.0)	10.0 (5.0–22.5)	0.648
Male/female, *n*	15/9	11/10	0.493
PRISM score, mean ± SD	22.4 ± 5.2	16.4 ± 7.2	0.003
FOUR score, mean ± SD	8.3 ± 3.0	10.4 ± 3.1	0.023
PI median (IQR)	1.33 (1.16–1.83)	0.99 (0.8–1.1)	<0.001
RI median (IQR)	75.0 (0.67–0.82)	61.0 (0.59–0.67)	<0.001
Use of vasopressors, *n* (%)	20 (83.3)	13 (61.9)	0.11
Mechanical ventilation, *n* (%)	22 (91.7)	14 (66.7)	0.036
ICU stay (days), mean ± SD	9.0 ± 5.0	19.8 ± 16.4	0.008

*FOUR score, Full Outline of Unresponsiveness score; HR, hazard ratio; PI, pulsatility index; RI, resistive index; ICU, intensive care unit; PRISM III, pediatric risk of mortality assessment*.

**Table 5:** Value of PI and RI in predicting mortality.

	**PI**	**RI**
Cutoff	1.13	0.65
AUC	0.86 (95.0% CI: 0.75–0.97)	0.77 (95.0% CI: 0.63–0.91)
*p*	<0.001	0.002
Sensitivity	83.3%	79.2%
Specificity	81.0%	61.9%

*PI, pulsatility index; RI, resistive index; AUC, area under the curve*.

**Table 6:** Predictors of SAE in the studied patients.

**SAE predictors**	**Univariate analysis**		**Multivariate analysis**	
	**OR (95% CI)**	***p***	**OR (95% CI)**	***p***
Age	1.0 (0.83–1.21)	0.87	–	–
PRISM score	1.09 (1.02–1.2)	0.009	1.1 (1.02–1.24)	0.004
Use of vasopressors	1.7 (1.1–2.3)	0.01	1.59 (1.14–2.6)	0.11
Pulsatility index	1.2 (1.1–1.45)	0.031	1.4 (1.1–1.64)	0.017
Resistive index	0.35 (0.16–0.8)	0.012	0.36 (0.11–0.68)	0.007

*PRISM III, pediatric risk of mortality assessment; SAE, sepsis-associated encephalopathy*.

**Table 7:** Predictors of mortality in the SAE children.

**Mortality predictors**	**Univariate analysis**			**Multivariate analysis**		
	HR^*^	**95% CI**	***p***	HR^*^	**95% CI**	***p***
Age	1.25	0.62–2.5	0.44	–	–	–
PRISM score	3.1	1.47–6.79	0.002	0.04	0.0–0.067	<0.001
FOUR score	1.1	1.00–1.22	0.043	1.08	0.95–1.32	0.16
PI	1.68	1.36–2.29	<0.001	1.32	0.92–1.86	0.18
RI	1.1	1.02–1.17	<0.001	1.35	1.18–1.49	<0.001

*FOUR score, Full Outline of Unresponsiveness score; HR, hazard ratio; PI, pulsatility index; RI, resistive index; ICU, intensive care unit; PRISM III, pediatric risk of mortality assessment; SAE, sepsis-associated encephalopathy*.

## Discussion

The classic neurologic injury associated with sepsis is septic encephalopathy, described as a reversible dysfunction of the central nervous system that can have a wide spectrum of clinical presentations from stupor and unresponsiveness to severe agitation and irritability (2). Alteration in cerebral autoregulation together with microcirculatory alterations could explain local hypoperfusion in absence of severe systemic hypotension. Several potential causes of impaired cerebral autoregulation during sepsis have been identified, including nitric oxide accumulation, blood–brain barrier breakdown due to neuro-inflammation, and impaired microcirculation (13).

The association between severe sepsis and septic shock and increased cerebral vascular resistance as assessed by TCD estimated PI and RI was previously reported (14). The present study further showed that pediatric septic patients with SAE had significantly higher PI and RI than had their counterparts without SAE. These findings are supported by the conclusions of Schramm et al. (24), who used TCD to assess cerebrovascular autoregulation in critically ill adult patients with severe sepsis or septic shock in association with sepsis-associated delirium (SAD). They noted significant association between impaired cerebrovascular autoregulation at day 1 of ICU admission and subsequent development of SAD (24).

Another study on adult septic patients identified TCD-derived PI in the first post-admission day as significant predictor of subsequent SAE with good sensitivity and specificity (25). Moreover, the study of (13), which recruited 100 adult patients with sepsis, reported a significant association between sepsis-associated brain dysfunction and altered cerebral autoregulation detected by TCD (13).

In the present study, PI was negatively correlated to FOUR score with high significance (*p* = 0.016). This means that cerebrovascular resistance (CVR) increases with the deepening of coma (the decline in FOUR score). Similar results are seen in different studies that show higher CVR with more disturbance of consciousness or delirium (26, 27).

PI was related to illness severity as estimated by PRISM III. This is unlike previous adult data by Pierrakos et al. (25), which showed that patients with high PI on the first day of ICU admission also had a lower Glasgow coma scale (GCS) at the initiation of sepsis with no relation to illness severity by Acute Physiology and Chronic Health Evaluation (APACHE) II score. However, unlike our group of children, not all the adults in their study have severe sepsis or septic shock.

The overall mortality rate in the present study is 32.0%. This figure is relatively high when compared with the 10.5 and 24.9% mortality rates reported by the American and multinational studies of Gorgis et al. (27) and Fitzgerald et al. (28), but it is fairly lower than the 50.0% mortality rate reported by the study of Nyirasafari et al. (29) from Rwanda (27–29). However, our mortality rate accords with the Chinese figure of 29.6% (30). The current study found that PI and RI values were higher in the non-survivor group in comparison with the survivor one. Other studies showed the same link between the higher CVR and mortality (25, 31).

Sanz et al. performed brain imaging of 49 pediatric septic shock patients, and the most frequent acute brain lesion patterns, found on neuroimaging, were ischemia and cerebritis (i.e., cerebral edema/damage in the clinical context of infection) (32). Sandquist et al. studied neuroimaging from septic pediatric patients focusing on long-term abnormalities rather than acute changes. The most common abnormal finding was volume loss (39%), which could be the final evolution from previous lesions, including ischemia or infarctions (33). This would go with our finding of decreased cerebral blood flow with the high PI and RI.

### Study Limitation

It would be better if patients were followed up after planned time period or intervention to better understand the influencing factors for changes on the cerebral hemodynamics.

## Conclusion

In cases of SAE, CVR is high and is associated with increased mortality. Future research on the possible effect of therapeutic interventions (e.g., hypertonic saline) in critically sick septic shock children for reduction of intracranial pressure and its impact on improving cerebral vasoconstriction may be beneficial. This may reduce the morbidities in septic shock survivors.

## Data Availability Statement

The raw data supporting the conclusions of this article will be made available by the authors, without undue reservation, to any qualified researcher.

## Ethics Statement

This study was approved by the Ethics committee of Cairo University, Egypt [I-041014]. Written informed consent to participate in this study was provided by the participants' legal guardian/next of kin.

## Author Contributions

HA created the idea creator and supervised the work. SE edited the paper and supervised the work. AG collected and analyzed patient data and submitted the paper. RH was the radiologist who revised the sonographic data. AB was the radiologist who measured the indices. AE designed the study. All authors contributed to the article and approved the submitted version.

## Conflict of Interest

The authors declare that the research was conducted in the absence of any commercial or financial relationships that could be construed as a potential conflict of interest.
